# The societal impact of implementing an at-home blood sampling device for chronic care patients: patient preferences and cost impact

**DOI:** 10.1186/s12913-022-08782-w

**Published:** 2022-12-15

**Authors:** Deon Lingervelder, Michelle M. A. Kip, Eva D. Wiese, Hendrik Koffijberg, Maarten J. Ijzerman, Ron Kusters

**Affiliations:** 1 Health Technology and Services Research Department, Technical Medical Centre, University of Twente, Enschede, The Netherlands; 2 Laboratory for Clinical Chemistry and Haematology, Jeroen Bosch Hospital, s-Hertogenbosch, The Netherlands

**Keywords:** Chronic diseases, Phlebotomy, At-home blood-sampling, Cost-minimization analysis

## Abstract

**Background:**

Diabetes mellitus, cardiovascular diseases, chronic kidney disease, and thyroid diseases are chronic diseases that require regular monitoring through blood tests. This paper first investigates the experiences of chronic care patients with venipuncture and their expectations of an at-home blood-sampling device, and then assesses the impact on societal costs of implementing such a device in current practice.

**Methods:**

An online survey was distributed among chronic care patients to gain insight into their experience of blood sampling in current practice, and their expectations of an at-home blood-sampling device. The survey results were used as input parameters in a patient-level monte carlo analysis developed to represent a hypothetical cohort of Dutch chronically ill patients to investigate the impact on societal costs compared to usual care.

**Results:**

In total, 1311 patients participated in the survey, of which 31% experience the time spent on the phlebotomy appointment as a burden. Of all respondents, 71% prefer to use an at-home blood-sampling device to monitor their chronic disease. The cost analysis indicated that implementing an at-home blood-sampling device increases the cost of phlebotomy itself by €27.25 per patient per year, but it reduces the overall societal costs by €24.86 per patient per year, mainly due to limiting productivity loss.

**Conclusions:**

Patients consider an at-home blood-sampling device to be more user-friendly than venous phlebotomy on location. Long waiting times and crowded locations can be avoided by using an at-home blood-sampling device. Implementing such a device is likely cost-saving as it is expected to reduce societal costs.

**Supplementary Information:**

The online version contains supplementary material available at 10.1186/s12913-022-08782-w.

## Background

In 2018, approximately 58% (~ 9.9 million people) of the Dutch population were diagnosed with at least one chronic disease [1]. More specifically, ~ 1.2 million people suffer from diabetes mellitus (DM) [2], ~ 1.6 million from cardiovascular diseases (CVD) [3], ~ 1,7 million from chronic kidney disease (CKD) [4], and ~ 0,6 million from thyroid diseases (TD) [5–8]. The prevalence of all these diseases increases with age, with 95% of the people above the age of 75 years suffering from at least one chronic disease [1]. The number of people diagnosed with a chronic disease will further increase due to aging of the Dutch population resulting in a larger chronic disease burden [9]. In current practice, patients with DM, CVD, CKD or TD are monitored between one to four times a year through blood testing [10–13]. Venipuncture, the process of obtaining intravenous access to collect blood, is an invasive procedure that can cause pain, distress and anxiety to patients [14, 15]. Besides the fact that phlebotomy is experienced as inconvenient, it is also accompanied by high healthcare costs in the case of DM, CVD, CKD and TD patients due to the large number of patients that need to be monitored repeatedly [16, 17].

Self-management is becoming increasingly important in healthcare [18]. It can reduce unscheduled care by improving disease control and quality of life, potentially reducing costs, and improving healthcare outcomes as a result [19]. At-home blood-sampling empowers patients by allowing them to take more control of their own healthcare [20]. Patients have control over where and when they want to perform the blood sampling, which reduces the possible disruption of their daily routines. Importantly, blood does not have to be sampled via venipuncture but can be collected with a finger prick, which is less invasive [19]. It has been shown that patients prefer a finger prick over venipuncture [21, 22], mainly since it is experienced as being less painful, although contradictory results have also been reported [23]. In point-of-care (POC) testing, blood is drawn at home by the patients themselves (typically with a finger prick) and tested immediately. The main drawback of POC tests in self-management is that the devices are often expensive (especially for a patient to purchase themselves) and that the diagnostic accuracy can be lower than the reference laboratory tests [24, 25].

A novel blood collecting device is Hem-Col (designed by Labonovum, Limmen, The Netherlands). This is a microtube which enables patients to sample their own blood via a finger prick, and send the blood sample through postage to the hospital or laboratory for analysis. The Hem-Col device allows reliable measurement after days of storage, resulting in a larger time frame for laboratories to analyze the sample [19]. Hem-Col tubes have the size of regular blood collection tubes, which makes analyzing by standard laboratory equipment possible [19]. Hem-Col is available to consumers outside hospital laboratories but is not yet implemented in current clinical practice. Implementing Hem-Col in clinical practice would allow physicians to order Hem-Col for patients when blood testing is needed.

An at-home blood sampling device as an alternative to venipuncture on location, has the potential to improve current practice for patients by saving time and introducing a more preferred sampling method. Given that only the sampling method and location are different, and that the analysis of the blood sample remains unaffected, the use of at-home blood sampling will, by definition, not affect patient health outcomes directly. However, it has the potential to improve convenience for patients, which has been shown to be an important aspect in overall healthcare delivery [26, 27]. Therefore, the aim of this paper is twofold. Firstly, to gain insight into how chronic care patients experience current practice (venipuncture on location) to monitor their disease and their expectations of and willingness to use an at-home device (specifically Hem-Col) for blood sampling as an alternative to current practice. Secondly, this paper also aims to perform a cost-minimisation analysis to investigate the impact on the societal costs if Hem-Col (as a realistic example of an at-home blood-sampling device) is implemented as compared to current practice. Although there are other aspects relevant to the actual implementation of an at-home blood sampling device, including potential organizational barriers, reimbursement of the device or safety concerns, this paper will focus only on patient preferences and societal costs.

## Methods

A survey was used to investigate patients’ recent experiences with venipuncture, their expectations of an at-home device, and their willingness to use such a device (specifically Hem-Col). A patient-level Monte Carlo analysis was performed to quantify the impact of implementing the Hem-Col device in clinical practice on the costs (from a societal perspective) using the survey results for some of the input parameters. The decision tree and probabilities are provided in Additional File 3. As this is not an analytical study, the accuracy and reliability of Hem-Col (finger-prick) blood samples as compared with venous blood samples were not examined. Instead, it is assumed that the Hem-Col blood samples will render the same results as venous blood samples, which is also indicated by the manufacturer (Labonovum).

### Survey

To gain insight into the patients’ perceptions of phlebotomy, semi-structured qualitative interviews were held with ten DM patients (by EDW). Two of these patients were also diagnosed with TD. A copy of the semi-structured interview questions can be found in Additional File 1. The findings from these qualitative interviews were used to design the final survey, consisting of 32 questions divided into four sections. Sections one and two comprised of questions to gather information on the patient’s demographic factors (Section one), and the patient’s current chronic disease(s), and details on their phlebotomy appointments (Section two). In Section three, patients were asked questions about their experience and preferences of phlebotomy appointments, while Section four introduced Hem-Col and asked about their expectations of the device. The full survey (translated to English) can be found in Additional File 1.

The survey was designed in Qualtrics XM and a link to the survey was distributed among several Dutch patient associations and Facebook support groups for patients with DM, CVD, CKD, or TD. The Dutch patient associations distributed the survey link via email to patients, while a link to the survey was posted on the applicable Facebook groups. To participate in the survey, patients had to be 18 years or older, must be diagnosed with at least one of the chronic diseases, and must receive blood testing to monitor the disease at least once a year. All responses were anonymous and only surveys that were fully completed were included for analysis.

Several survey outcomes were used as input parameters for the cost-minimisation analysis, namely, the number of phlebotomy appointments per year, the appointments' location, the time spent per appointment (including travel time), the dependency on others, and the willingness to use Hem-Col.

To investigate whether the survey respondents are representative of all DM, CVD, CKD, or TD patients in the Dutch population, the distribution of respondents among Dutch provinces, as well as their age and gender were compared with the Dutch population. To determine whether a statistical significant difference exists between the respondents and the Dutch population, a χ2-test was performed for gender and distribution among the provinces, and a one-sample t-test was performed for age.

### Cost minimisation analysis

A patient-level Monte Carlo analysis was developed in Excel to represent the Dutch population that is suffering from DM, CVD, CKD, or TD. This analysis allows to estimate how the distribution of input parameters affects the distribution of the final results [28]. No actual simulation model was developed and used, since the aim was not to extrapolate beyond the current evidence base. Each hypothetical patient was assigned a gender and an age, based on data from the literature. Each patient was also assigned one (or multiple) of the four chronic diseases with a chance dependent on the age of the patient. It was assumed that all these patients are eligible for using Hem-Col. Although it has been shown that finger prick (i.e. capillary) sampling is more likely to lead to sampling errors than venous sampling [29, 30], the Hem-Col device is designed to avoid sampling errors by providing clear instructions on how to accurately sample blood via a finger prick and how to correctly package the sample when sending it via post. Nonetheless, a 5% sampling error rate was incorporated into the analysis, indicating that 5% of patients that uses Hem-Col will need to provide a new sample.

The primary outcome measure was the incremental societal costs when implementing Hem-Col (for at-home blood sampling) as compared with current practice (on-site blood sampling). The health effects are assumed to be negligible since the tests performed and therefore test results will remain the same whether Hem-Col is used or not, and Hem-Col will therefore not have any direct health effects in the long term. All costs were evaluated from a societal point of view, over a time horizon of one year. No discount rate was applied due to the time horizon.

### Costs

The volume of the blood sample does vary and a fixed volume of buffer is added to the Hem-Col tube at the laboratory. Therefore, the tubes contain lithium as an internal standard that is measured to calculate the dilution factor. The costs of the dilution and examination were calculated by taking the average cost of three Dutch laboratories. The tariff for the order of the blood tests, which is the same for all phlebotomy locations and Hem-Col, and the costs of shipment by mail were also added to the Hem-Col costs. The selling price of the Hem-Col device was provided by the manufacturer.

In the Netherlands, phlebotomy can be performed at four potential locations, namely the hospital, a service phlebotomy center, the general practitioner (GP) or at home. The costs for a phlebotomy appointment at the hospital were calculated by taking the average reimbursement tariff of five hospitals, while the costs for the other locations were calculated by taking the average tariff per location as provided by ten large laboratories.

Additional potential costs that were taken into account include travel, parking, productivity losses, and time spent by an informal caregiver. An overview of these costs is provided in Table 1. Traveling costs, parking costs and costs of productivity loss were derived from the Dutch Costing Manual [31]. With Hem-Col, traveling costs were seen as all costs associated with mailing the sample to the laboratory, including travel and postage. Full productivity loss costs were accounted for until the age of 65. Approximately 12.1% of the Dutch population remain in employment after the age of 65 and 1.8% after the age of 75 [32]. The productivity loss costs for these age groups were calculated by multiplying the full productivity loss costs with these percentages. Furthermore, patients may be dependent on others for the phlebotomy appointment resulting in additional productivity loss costs of an informal caregiver.

**Table 1:** Costs overview

Parameter	Category	Expected value	95% CI^a^
Phlebotomy Cost	Hospital	€ 9.04	€8.08 to €9.99
	Phlebotomy service center	€ 15.34	€14.09 to €16.60
	GP's office	€ 18.13	€17.92 to €18.34
	At home	€ 25.16	€19.36 to €30.96
	Hem-Col	€ 20.42^b^	€10.42 to €30.43
Traveling Cost	Hospital	€ 6.08	€3.10 to €9.07
	Phlebotomy service center	€ 1.02	€0.52 to €1.52
	GP's office	€ 0.45	€0.23 to €0.67
	Hem-Col	€ 1.02	€0.52 to €1.52
Productivity Lost Cost per Hour	Male age 18–64	€ 40.74	€20.78 to €60.70
	Male age 65–74	€ 4.91	€2.51 to €7.32
	Male age 75 +	€ 0.73	€0.37 to €1.09
	Female age 18–64	€ 33.97	€17.32 to €50.61
	Female age 65–74	€ 4.10	€2.09 to €6.10
	Female age 75 +	€ 0.61	€0.31 to €0.91
	Informal care giver	€ 15.05	€7.68 to €22.42
Waste Cost	Regular tube *(per 100 tubes)*	€ 1*.*22	€0*.*62 to €1*.*82
	Hem-Col tube *(per 100 tubes)*	€ 0*.*68	€0*.*35 to €1*.*01

*GP* General Practitioner

^a^based on normal distribution for the mean and corresponding standard error

^b^includes cost of the test and tube, postage and shipping to patient, dilution factor and order tariffs

After analysis the Hem-Col tube can be split into two parts, an upper and lower part. The upper part contains the blood sample, and the lower part contains no blood, meaning it can consequently be disposed of as residual waste, which is less expensive to process than medical waste [33]. The medical waste costs were derived by taking the average of two waste process organizations: Renewi and Suez.

All costs are provided in Euros and were converted to 2020 prices using consumer price indices (CPI) provided by Statistics Netherlands [34].

### Multiple diseases

No data could be found in the literature on the distribution of the patients with multiple of the chronic diseases included in this study (DM, CVD, CKD, TD) across the Dutch population, nor information on the age range or gender distribution of these patients. Therefore, the gender distribution, age distribution and the risk of a patient having multiple chronic diseases (per age group) were calculated as an average of the relevant data found for DM, CVD, CKD and TD.

### One-way sensitivity analysis and probabilistic analysis

A one-way sensitivity analysis was performed to determine the effect of individual parameters on the cost outcome. Monte Carlo simulations were performed for a probabilistic analysis, using 10,000 iterations of 100,000 hypothetical chronic care patients with one of the four chronic diseases or multiple diseases. All input parameters were represented by a distribution to acquire probabilistic values and 95% confidence intervals. An overview of all parameters is provided in Additional File 3.

### Scenario analysis

A scenario analysis was performed to investigate what the cost of the Hem-Col device should be to ensure that the overall phlebotomy cost when implementing Hem-col, remains comparable to current practice (venipuncture on location).

## Results

### Survey responses

There were 1363 patients that completed the survey, of which 1311 patients were included in the analysis. Eleven patients were excluded since they did not want to participate and 41 patients were outside of the target group. The biggest patient groups are CVD (28%) and TD (26%), while patients with CKD, DM and multiple diseases make up 10%, 17% and 19% of the respondents.

From the χ2-test and the one-sample t-test (to investigate the representativeness of survey respondents to the Dutch population), it was found that in terms of gender and province only the respondents with CKD are representative of the Dutch population. Further results can be found in Additional File 2.

A summary of the patient characteristics is provided in Table 2. Of the responding chronic care patients, 449 (34%) were male. The mean age of the respondents was 54.3 years (SD = 15.9), the mean number of phlebotomy appointments per year was 4.4 (SD = 5.5), and the mean time spent per appointment including travel time was 1.1 h (SD = 0.5). Most patients visit the hospital for their phlebotomy appointment (50%), followed by the phlebotomy service center (40%) and the GP’s office (7%). Three percent of patients already makes use of at-home sampling. This was incorporated in the estimation of usual care costs.

**Table 2:** Patient Characteristics of Survey Participants

	**Total**	**DM**	**CVD**	**CKD**	**TD**	**Mult**
Participants [n(%)]	1311 (100%)	222 (17%)	369 (28%)	127 (10%)	345 (26%)	248 (19%)
Male [n(%)]	449 (34.2%)	77 (35%)	229 (62%)	31 (24%)	19 (6%)	93 (38%)
Age in years [mean(sd)]	54.3 (15.9)	45.9 (18.8)	64.5 (10.0)	45.5 (14.8)	48 (12.3)	59.6 (14.4)
Phlebotomy appointments per year [mean(sd)]	4.4 (5.5)	3.6 (3.6)	3.9 (6.6)	6.2 (4.2)	4.6 (3.5)	4.7 (4.1)
*Location*						
Hospital [n(%)]	656 (50%)	127 (57%)	137 (37%)	105 (83%)	159 (46%)	128 (52%)
Phlebotomy service center [n(%)]	523 (40%)	81 (36%)	174 (47%)	18 (14%)	151 (44%)	99 (40%)
GP's office [n(%)]	96 (7%)	9 (4%)	34 (9%)	3 (2%)	35 (10%)	15 (6%)
At home [n(%)]	36 (3%)	5 (2%)	24 (7%)	1 (1%)	0 (0%)	6 (2%)
Time spent per appointment, including travel time in hours [mean(sd)]	1.1 (0.5)	1.06 (0.5)	0.90 (0.5)	1.39 (0.60)^a^	0.97 (0.5)	1.11 (0.6)
Time spent seen as a burden [n(%)]	410 (31%)	84 (38%)	64 (17%)	50 (39%)	132 (38%)	80 (32%)
*Feeling before phlebotomy appointment*						
I don't care [n(%)]	959 (73%)	130 (59%)	295 (80%)	95 (75%)	250 (72%)	189 (76%)
I prefer not to go [n(%)]	189 (14%)	56 (25%)	39 (11%)	13 (10%)	48 (14%)	33 (13%)
Anxiety [n(%)]	163 (12%)	36 (16%)	35 (9%)	19 (15%)	47 (14%)	26 (10%)
*Venous blood sampling is painful*						
Yes [n(%)]	73 (6%)	17 (8%)	16 (4%)	6 (5%)	14 (4%)	20 (8%)
No [n(%)]	705 (54%)	105 (47%)	233 (63%)	59 (46%)	174 (50%)	134 (54%)
Sometimes [n(%)]	533 (41%)	100 (45%)	120 (33%)	62 (49%)	157 (46%)	94 (38%)
Feeling dependent on others [n(%)]	210 (16%)	36 (16%)	48 (13%)	21 (17%)	53 (15%)	52 (21%)
Affects their daily schedule [n(%)]	413 (32%)	75 (34%)	86 (23%)	49 (39%)	117 (34%)	86 (35%)

*GP* General Practitioner

^a^Four respondents were removed from the calculation of this parameter, since they included the time spent for dialysis in their survey response

The mean age of CVD-patients is 64.5 years, which is 12–15 years higher than the mean age of DM-, CKD- and TD-patients. Patients with CKD have the most phlebotomy appointments (± 6 times per year), visit the hospital most often for a phlebotomy appointment (83%) and spend the most time at the appointment including travel time (1.39 h) compared with the other chronic diseases. Most CVD-patients go to the phlebotomy service center for an appointment (47%) and they spend the least time per appointment including travel time (0.9 h). Compared with the other groups, fewer CVD-patients experience the time spend per appointment as a burden (17%) and fewer CVD-patients stated that the appointment affects their daily schedule (23%). More DM-patients prefer not to go to the appointment (25%) or experience anxiety (16%) compared with CVD-, CKD- and TD-patients. Most CVD-patients do not experience venous phlebotomy as painful (63%), while for the other groups, this is 54% or lower.

### Survey results

A detailed summary of the survey results can be found in Additional File 2. Of all responding patients, 71% are willing to use Hem-Col; 81% of this group wants to use it for all tests that monitor their chronic disease. The biggest motivator for patients to use Hem-Col was the ability to do the blood sampling themselves and that the blood sampling would take less time. Diabetes patients were most willing to use Hem-Col (85% of DM patients) while CVD-patients were the least willing to use Hem-Col (70% of CVD patients).

Of all responding patients, 35.1% preferred a finger prick, 21.7% preferred venous sampling, 36.7% had no preference, and 6.5% did not know. The preference for a finger prick was the highest among DM patients (45%) and the preference for venous sampling was lowest (15%) compared with other groups.

### Cost analysis

**Fig. 1:**
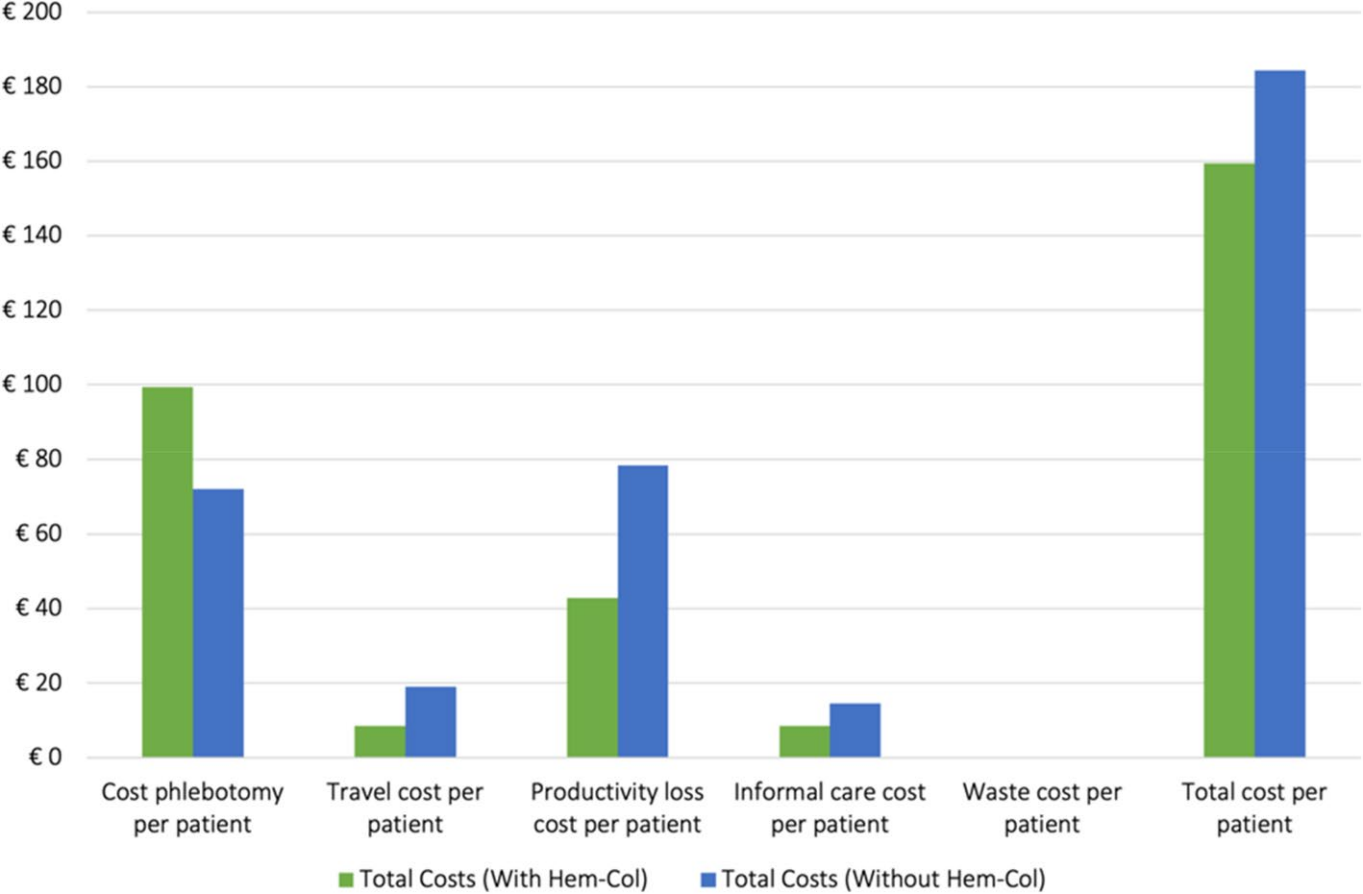
Analysis results of 100,000 hypothetical patients per year

The average outcomes of the PA samples are presented in Fig. 1. As seen in the Figure, the average cost savings are mainly due to a decreased time spent per phlebotomy appointment, resulting in a reduction of the productivity loss cost with €35.55 per patient per year. The results showed a negligible impact of the waste cost on the societal cost (€0.05), while the travel cost and informal care cost per patient per year decrease with €10.56 and €5.94, respectively. Although the cost of phlebotomy increases with €27.25 per patient per year when using Hem-Col, the overall societal cost (-€24.86 per patient per year) remains negative, indicating that the societal costs can be reduced when Hem-Col is implemented.

**Fig. 2:**
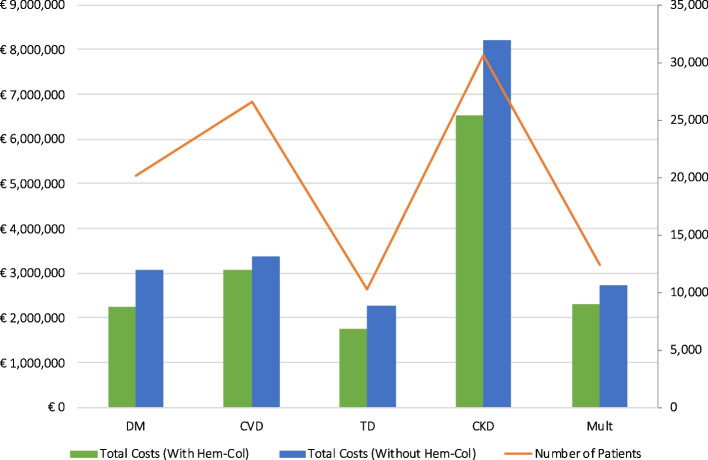
Summary of total costs per patient group

As illustrated in Fig. 2, the two largest patient groups were CVD (*n* = 26,608) and CKD patients (*n* = 30,556). Even though these two patient groups were both large, the total costs for CKD were much higher compared to CVD. This is mainly due to CKD patients having more phlebotomy appointments per year. The difference in costs when implementing Hem-Col versus without implementing Hem-Col is largest for TD and CKD patients, since these patients are, on average, younger and therefore have less productivity losses due to Hem-Col.

### Probabilistic analysis

**Fig. 3:**
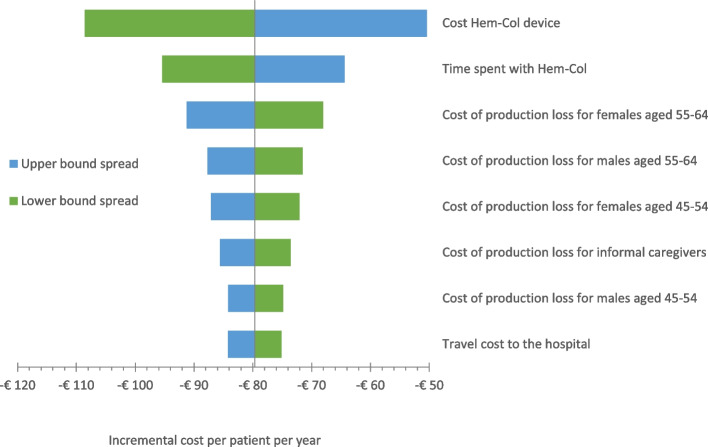
A tornado diagram showing the eight most influencing model parameters on the cost outcome

As seen in Fig. 3, the one-way sensitivity analysis shows that the cost of the Hem-Col device has the largest impact on the cost outcome due to parameter uncertainty, followed by the time spent when Hem-Col is used for blood sampling.

**Fig. 4:**
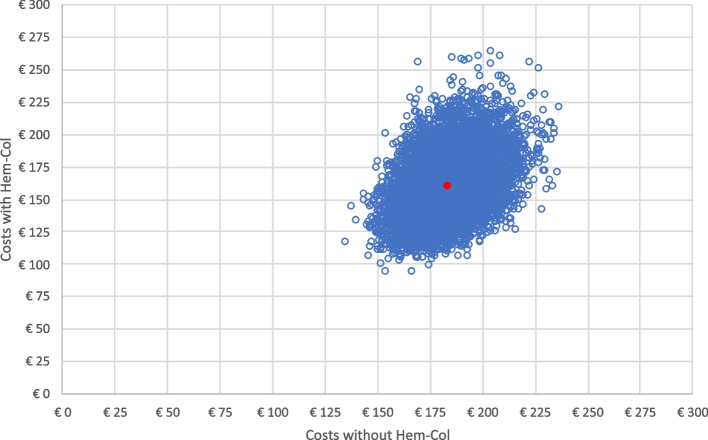
PA result of 10,000 iterations of 100,000 patients

The result of the PA is shown in Fig. 4, where the costs of current practice are plotted against the costs after implementing Hem-Col. The PA result indicates that phlebotomy with the possibility to use Hem-Col costs on average €159.44 (95% CI €119.10 to €208.35) per patient on a yearly basis, as compared with €184.30 (95% CI €159.08 to €212.53) for current practice, representing cost savings of €24.86 (95% CI -€39.98 to -€4.18) per patient per year.

### Scenario analysis

The results of the scenario analysis can be found in Additional File 3. It was found that the cost of hem-col should be €12.81 (opposed to the current cost of €22.42) for the phlebotomy costs to remain similar to that of venipuncture on location.

## Discussion

This study provided new insights into how patients experience venipuncture, their willingness to use an at-home blood sampling device such as Hem-Col, and the effect that such a device can have on societal costs. A significant number of chronic disease patients can be considered to adopt home-sampling devices that, from a societal perspective, are cost-saving and moreover positively affects the self-management of their disease. One-third of the patients diagnosed with DM, CVD, CKD, TD or multiple diseases experience the phlebotomy appointment as a burden and indicated that it affects their daily schedule. Approximately 46% of the patients reported physical inconveniences after venous phlebotomy. Additionally, 16% feel dependent on others while 12% reported anxiety in anticipation of phlebotomy appointments. The most important factors of dissatisfaction towards phlebotomy were accredited to long waiting times and crowded phlebotomy locations. The recent outbreak of COVID-19 is likely to have provoked further dissatisfaction towards long waiting times and crowded phlebotomy locations. Chronic care patients are at higher risk for COVID-19 complications and this could have led to further adversity towards crowded phlebotomy locations.

Based on the responses from chronic care patients, blood sampling devices' success depends on its safety and trustworthiness, clarity of instructions, ease of use, and ease of sending it in for testing. Although a consistent preference for a finger prick is lacking, approximately two out of three patients are interested in Hem-Col, with about 71% of respondents preferring to use Hem-Col to monitor their chronic disease. The most prevalent reason for patients’ indifference toward Hem-Col is attributable to the expectation of discomfort of self-administering the blood sampling. It has been shown that, in general, a finger prick is preferred over venous sampling, mainly due to patients experiencing a finger prick as less painful [21, 22, 35, 36]. In this study, the percentage of patients preferring a finger prick (35%) is higher than the percentage of patients preferring venous sampling (22%). The same proportion of patients who preferred a finger prick or venous sampling found their preferred form of sampling less painful, indicating that patients’ opinions vary within this topic. This could be since venipunctures are easier to perform on some patients compared to others.

The interest in using Hem-Col and patients' preference for finger prick instead of venous sampling varied between patient groups. Diabetes patients showed a higher interest in Hem-Col and had the highest preference for the finger prick sampling method compared with other patient groups. This can be explained by the reason they gave in the survey for preferring a finger prick: ‘being used to it.’ Diabetes patients are familiar with performing a finger prick to measure their blood glucose levels throughout the day. The preference for a finger prick was slightly lower among CKD patients compared to the other groups, which can be explained by the possibility of blood sampling during dialysis. When patients are on dialysis, the blood can be easily drawn without performing an extra venipuncture. CVD patients had the lowest interest in using Hem-Col, which could be due to their age. The mean age of respondents with CVD was approximately ten years higher compared with other groups. Older people are, in general, less eager to learn how to use a new system and prefer to use a system they are familiar with [37, 38]. Patients who are suffering from chronic diseases besides DM, CVD, CKD or TD had a lower willingness to use Hem-Col. This can be explained by the increased amount of hospital appointments for these patients, where phlebotomy is typically combined with another appointment. Consequently, for these patients, the impact of phlebotomy appointments on their daily schedule is less than that of other patients, and they may therefore value at-home blood sampling less.

Several limitations were perceived in this study. Firstly, other than testing for face validity, the survey was not tested for reliability or validity. Secondly, splitting input parameters into multiple categories resulted in a few very small subgroups. Performing analysis on these small subgroups resulted in high parameter uncertainty and, therefore, large 95% CI intervals for the cost outcomes. Thirdly, after analyzing the respondents' remarks at the end of the survey, some confusion among CVD patients was observed. For some CVD patients, it was not clear that Hem-Col cannot be used to examine their international normalized ratio (INR). Several CVD patients indicated their INR is tested with a finger prick and therefore, they did not see the added value of Hem-Col. Lastly, it is again important to note that in this study it was assumed that the diagnostic performance of Hem-Col would be identical to venous sampling. However, the inevitable risk with an at-home blood sampling device is the risk of a sampling error. Although this risk is minimized by detailed instructions provided along with the Hem-Col device, it is uncertain whether the assumed 5% sampling error rate adequately reflects clinical practice. Besides the additional costs of repeated blood sampling, the potential impact of such sampling error in terms of inaccurate or incorrect test results is unknown. However, however, the current analysis also conservatively overestimates the success of venous blood sampling performed by a phlebotomist, by assuming that no sampling errors occur with this method. Therefore, it is unlikely that the uncertainty in sampling errors will have changed the main findings. However, it should be acknowledged that a higher sampling error rate of Hem-Col decreases satisfaction among patients which may eventually reduce the willingness to use Hem-Col.

On average, patients were willing to pay €2.15 per phlebotomy appointment to use the Hem-Col device. The financial contribution that DM-patients were willing to make was the lowest among all patient groups, even though they had the highest preference to use Hem-Col. This could be since type 2 diabetes occurs more frequently in people with a lower socio-economic status and less purchasing power [39].

## Conclusions

Of the chronically ill patients, approximately 70% prefer to use Hem-Col for blood sampling to monitor their disease. Blood sampling with Hem-Col is considered more user-friendly compared with venous phlebotomy. Hem-Col may reduce the burden on patients, lower the impact of the phlebotomy appointment on their daily schedule, and reduce physical inconveniences. Long waiting times and crowded phlebotomy locations can be avoided when patients can self-manage using Hem-Col. Integrating an at-home blood sampling device with a telehealth program could further accentuate the benefits of both concepts. Furthermore, implementing Hem-Col to monitor chronic diseases is likely cost-saving compared with current practice as it is expected to reduce societal cost. The total cost saving per patient might seem small or limited, but when considering how large each of the patient groups is, the implementation of Hem-Col could have a substantial impact nationwide. Seeing as the willingness to use Hem-Col is different between subgroups, it would be useful to start with a small-scale implementation in one of the more willing groups (such as DM patients) before implementing across different disease areas. Furthermore, although the manufacturer has shown that the diagnostic performance of Hem-Col sampling is comparable with venous sampling in a laboratory setting, future research should also investigate whether the diagnostic performance remains comparable when patients perform the Hem-Col sampling themselves. Although Hem-Col will reduce costs from a societal perspective, the same can not be said for the healthcare system perspective. The most significant impact on costs was the reduced productivity loss costs, meaning foremost patients and their employers will benefit from implementing an at-home sampling device. This comes at the expense of the healthcare system (that is, at the expense of all Dutch citizens together funding the reimbursements through this system) due to the increased phlebotomy costs. As found in the scenario analysis, the cost of the Hem-col device would have to decrease by 37.27% to be comparable with the costs of venipuncture on location. That said, the current cost of the Hem-col device is a starting price and is likely to be reduced when Hem-Col is used on a larger scale. This will result in lower phlebotomy costs for Hem-Col and therefore larger cost-savings when Hem-Col is implemented in clinical practice.

## Supplementary Information

**Additional file 1.** Interview & Survey.

**Additional file 2.** Survey results.

**Additional file 3.** Details of the cost-minimisation analysis.

## Data Availability

The datasets generated during and/or analysed during the current study are available from the corresponding author on reasonable request.

## Abbreviations

CKD: Chronic Kidney Disease
CPI: Consumer Price Index
CVD: Cardiovascular Disease
DM: Diabetes Mellitus
GP: General Practitioner
INR: International Normalised Ratio
PA: Probabilistic analysis
POC: Point-of-Care
SD: Standard Deviation
TD: Thyroid Disease
