# Supplementary material for: The societal impact of implementing an at-home blood sampling device for chronic care patients: patient preferences and cost impact
Source: BMC Health Serv Res. 2022 Dec 15;22:1529. doi: 10.1186/s12913-022-08782-w (PMC9753888; doi:10.1186/s12913-022-08782-w)
Supplement: Supplementary file 1 — Additional file 1. Interview & Survey. [file 12913_2022_8782_MOESM1_ESM.docx]

# Additional File 1: Interview & Survey

To design the survey, EDW held semi-structured qualitative interviews with ten DM patients, two of whom were also diagnosed with TD, to gain insight into the patients’ perceptions of phlebotomy. The survey was tested for face validity by (some of) the co-authors and an informal pilot test was done with 7 healthy volunteers. No other validity or reliability tests were performed.

The survey was designed on Qualtrics XM and distributed among several Dutch patient associations and Facebook support groups for patients with DM, CVD, CKD, or TD. The Dutch patient associations distributed the survey via email to patients, while a link to the survey was posted on the applicable Facebook groups.

The semi-structured interview questions and the survey can be found below.

## Semi-structured interviews

**Introduction**

Welcome to this interview, I will first introduce myself. I am a master’s student of Health Sciences at the University of Twente. I am doing research on how we can decentralize blood collection, or in other words the possibility to take blood at home. The focus here is on people who need to have blood taken regularly (several times a year). The purpose of this interview is to gain more insight into the patient's perspective. After the interviews, a survey will be made, this way the survey will be more in line with the target group. There are no wrong answers, I am interested in your opinion. This interview will take approximately 20 to 30 minutes.

I would like to remind you that participation in this interview is voluntary, you may refuse to answer any question or stop the interview at any time without reason or explanation on your part. The answers you provide will be used anonymously in this study. In addition, an audio recording of this interview will be made.

Do you give your permission for the things I have just mentioned?

Do you have any questions for me before we begin?

**General**

What is your age?

What chronic disease do you suffer from that requires regular blood sampling?

How many times a year do you need to take blood samples for monitoring this chronic disease?

How much time does this blood collection take each time, including travel time to the location?

How do you experience the regular (venous) blood collection (discomfort, loss of time, a burden)?

Does the blood draw have an impact on your daily routine? If so, what causes it to have an impact?

Is it usually easy or difficult to get a blood sample from you?

A new blood collection system is currently being developed. This allows patients to draw blood at home by finger pricking. The blood is then collected in a small tube, after which it can be sent by mail.

What do you think about if blood collection could be done at home?

What do you expect from such a system, or what do you find important in such a system? (Easy to use, fast, reimbursement by health insurance etc?)

What do you think are the advantages and disadvantages of such a system? (Time/ease, convenient, amount of drops needed?)

Would you like to use such a system? Why or why not?

How often do you expect to use this system?

Do you have a preference for a finger prick instead of the venous collection?

**Closing**

We have come to the end of this interview. Do you have any questions or things you would like to add to this interview?

Thank you for your participation.

If you have any questions or comments at a later time, please contact me by email or by phone.

## Survey

Welcome,

This research focuses on at-home phlebotomy possibilities for chronically ill patients. The purpose of this survey is to gain insight into your perspective. You are requested to answer a few questions about your experiences with phlebotomy appointments in the hospital or at a service phlebotomy center. Afterwards, we will ask you about your opinion of a new system that enables blood sampling at home.

It will take approximately 10 minutes to complete the survey. The answers you provide will be processed anonymously and cannot be traced back to you. Your participation in this survey is voluntary. You have the right to quit the survey at all times without giving a reason and this will not lead to negative consequences for you. The results are owned by the University of Twente and will only be used for scientific purposes. In case you have any questions, feel free to contact the researcher via e-mail (…).

By clicking the button below, you agree to participate on voluntary basis to this research, you are at least 18 years old and you are aware of the possibility to withdraw from the survey at any time without giving a reason.

Thank you in advance for your time and effort.

- I agree, start the survey
- I do not agree, I wish not to participate

*In case “I do not agree, I wish not to participate” was chosen, the survey ends.*

1. What is your age?

___________________

2. What is your gender?

- Male
- Female
- Different

3. In what province do you live? Please, indicate in the figure.


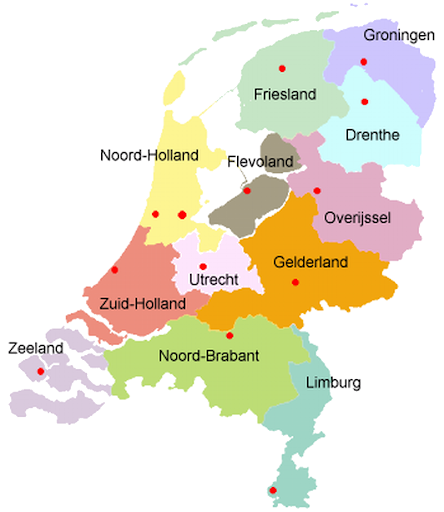


4. With which chronic disease are you diagnosed that your blood needs to be tested on a regular basis? (Multiple answers are possible)

- Diabetes mellitus type 1
- Diabetes mellitus type 2
- Cardiovascular disease
- Chronic kidney disease
- Thyroid disease
- Different, namely:

___________________

5. How many phlebotomy appointments do you have per year to monitor this chronic disease?

- 1 appointment
- 2 appointments
- 3 appointments
- 4 appointments
- 5 appointments
- 6 appointments
- More than 6 appointments

*In case “More than 6 appointments” was chosen, the survey continues with question 6, otherwise question 6 was skipped.*

6. You indicated to have more than 6 phlebotomy appointments per year, how many appointments do you have?

___________________

7. Which location do you visit the most for phlebotomy?

- The hospital
- The service phlebotomy center
- The general practitioners office
- Phlebotomy appointments often take place at home

8. How much time do you spent per phlebotomy appointment, including travel time from and to the location?

- Less than half an hour
- Half an hour till an hour
- An hour till one and a half hour
- One and a half hour till two hours
- More than two hours, namely:

___________________

9. Is the time spent per phlebotomy appointment a burden to you?

- Yes
- No

10. How do you feel when you think about the fact that your blood must be drawn venously?

- I don’t care
- I don’t feel anxiety, but I prefer not to go
- I feel anxiety

*In case “I feel anxiety” was chosen, the survey continues with question 11, otherwise question 11 and 12 were skipped.*

11. How much anxiety do you experience before a phlebotomy appointment? 0 indicates that you feel no anxiety at all and 10 indicates that you feel an extreme amount of anxiety.


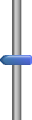
10

0

12. Do you fear needles?

- Yes
- No

13. Would you describe venous phlebotomy as painful?

- Yes
- No
- Sometimes

14. Do you feel dependent on others to go to the hospital, the service phlebotomy center or the GP’s office for the phlebotomy?

- Yes
- No

*In case “Phlebotomy appointments often take place at home” was chosen in question 7, question 15 was skipped.*

15. How do you experience the phlebotomy at your chosen location (hospital, service phlebotomy center or the GP’s office)?

|  | Strongly agree | Agree | Neither agree nor disagree | Disagree | Strongly disagree |
| --- | --- | --- | --- | --- | --- |
| I experience the waiting room as unpleasant |  |  |  |  |  |
| It is hard to find a parking spot |  |  |  |  |  |
| It is always busy |  |  |  |  |  |
| I have to wait for a long time |  |  |  |  |  |
| The blood sampling itself takes a long time |  |  |  |  |  |
| The phlebotomist is unfriendly |  |  |  |  |  |
| The phlebotomist is not good at her job |  |  |  |  |  |

16. After blood is drawn from a vein … (multiple answers possible)

- I often get bruises
- I bleed frequently
- I have muscle pain
- I feel lightheaded
- I pass out sometimes
- None of the above

17. Does the phlebotomy appointment affects your daily schedule, besides the phlebotomy itself and the travel time?

- Yes
- No

*In case “Yes” was chosen, the survey continues with question 18, otherwise question 18 was skipped.*

18. How does the phlebotomy appointment affects your daily schedule?

_________________________________________________________________________________________________________________________________________________________________________________________________________________________________________________________________________________

19. What is your preference, based only on the blood-sampling method itself; a finger prick or venous sampling? We request you to base your preference only on the method itself, and not on the location where the blood-sampling can take place (e.g. at home/hospital).

- A finger prick
- Venous sampling
- I don’t have a preference
- I don’t know

*In case “A finger prick” was chosen, the survey continues with question 20. In case “Venous sampling” was chosen, the survey continues with question 21. In case “I don’t know” was chosen, the survey continues with question 22. In case “I don’t have a preference” was chosen, the survey continues with question 23.*

20. Why do you have a preference for the finger prick? (Multiple answers possible)

- It is less painful
- I am used to a finger prick
- It is quicker
- It is easier
- My veins are hard to find
- I have a fear of needles
- The bleeding stops sooner
- No bruises
- No muscle pain
- Different, namely:

___________________

*The survey continues with question 23.*

21. Why do you have a preference for venous sampling? (Multiple answers possible)

- It is less painful
- I am used to venous sampling
- I don’t have to do it myself
- It seems uncomfortable to get enough blood in a tube after a finger prick
- Different, namely:

___________________

*The survey continues with question 23.*

22. Did you ever use a finger prick, or someone else on you?

- Yes
- No

23. A new blood-sampling system has been developed which makes it possible for patients to do the blood-sampling themselves at home. The package will be send via mail and contains the following items (see the figure):

- A sterile cloth
- A lancet to prick the finger
- Tube to collect the blood
- A band aid
- Shipping material

The patient does the finger prick him-/herself, or someone nearby who is willing to help. After the prick, the blood can be collected in the tube. Approximately 5 blood drops are needed to fill the tube. Finally, the package can be send via mail.


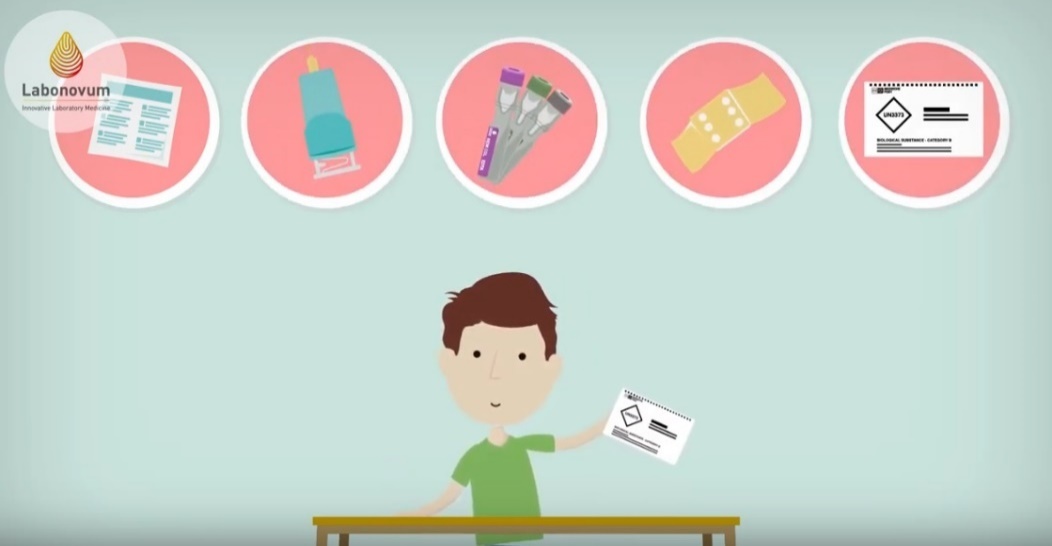


What do you think when you are able to do the blood-sampling at home with a finger prick?

- Great
- Good
- Average
- Not good
- Terrible

*In case “Great” or “Good” was chosen, the survey continues with question 24. In case “Not good” or “Terrible” was chosen, the survey continues with question 25. In case “Average” was chosen, the survey continues with question 26.*

24. For what reasons are you interested in blood-sampling at home with a finger prick? (Multiple answers possible)

- I can do it myself
- It is easier to schedule
- It takes less time
- I don’t have to travel back and forth
- Different, namely:

___________________

*Survey continues with question 26.*

25. For what reasons are you not interested in blood-sampling at home with a finger prick? (Multiple answers possible)

- I think it is a hassle to do it myself
- I am afraid to prick myself
- I see it as a trip to go to the hospital, the service phlebotomy center or the GP’s office for phlebotomy
- Different, namely:

___________________

26. What do you think is important for a blood-sampling system usable at home? 5 Stars indicate that you think it’s extremely important, 1 star indicates that you think it’s not important.

|  |  |
| --- | --- |
| It must be easy to use | 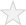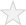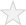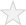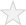 |
| It must be quick | 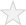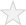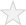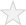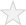 |
| It must be less painful than venous sampling | 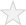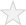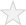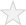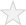 |
| The system must be safe and trustworthy | 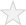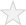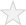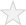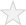 |
| The system must be usable anywhere | 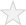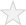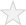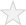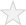 |
| It must contain clear instructions | 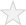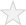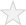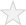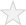 |
| It must be easy to send to the laboratory | 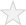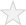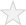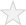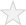 |
| The lancet must be adjustable in height | 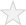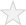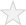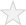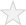 |
| Only a small amount of blood is required | 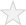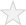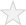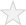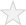 |

27. A blood collection tube needs approximately 5 blood drops, do you think that is a lot?

- No, that’s fine
- Yes, I think that is a lot but it’s still doable
- Yes, that seems hard to me

28. How much are you willing to spend on an additional contribution to be able to do the blood-sampling at home? The costs mentioned are per phlebotomy appointment.

- Nothing
- €5,00
- €10,00
- €20,00
- €30,00
- I am willing to pay more than €30,00

*In case “I am willing to pay more than €30,00” was chosen, the survey continues with question 29, otherwise question 29 was skipped.*

29. You indicated that you are willing to pay more than €30,00 on an additional contribution to be able to do the blood-sampling at home. How much are you willing to pay?

___________________

30. Are you willing to use this system, assuming that the costs are not higher than the additional contribution you are willing to pay? For example: If you have answered with €5,00 ; the costs of the system will not be higher than €5,00.

- Yes
- No

*In case “No” was chosen, the survey continues with question 32.*

31. How often do you want to use this system?

- For all blood tests I do on a yearly basis to monitor my chronic disease
- For a part of the blood tests I do on a yearly basis to monitor my chronic disease, amount:

___________________

32. Do you have any remarks?

_________________________________________________________________________________________________________________________________________________________________________________________________________________________________________________________________________________

Thank you for your participation, your answers have been recorded.
