# Supplementary material for: The societal impact of implementing an at-home blood sampling device for chronic care patients: patient preferences and cost impact
Source: BMC Health Serv Res. 2022 Dec 15;22:1529. doi: 10.1186/s12913-022-08782-w (PMC9753888; doi:10.1186/s12913-022-08782-w)
Supplement: Supplementary file 2 — Additional file 2. Survey results. [file 12913_2022_8782_MOESM2_ESM.docx]

# Additional File 2: Survey results

## Summary of survey results

Additional Table 1. Reasons for having a preference for a particular sampling method.

|  | Total | DM | CVD | CKD | TD | Mult |
| --- | --- | --- | --- | --- | --- | --- |
| *Preference for blood-sampling* | | | | | | |
| Finger Prick | 460 (35%) | 100 (45%) | 120 (33%) | 33 (26%) | 106 (31%) | 101 (41%) |
| I don’t know | 85 (6%) | 6 (3%) | 27 (7%) | 15 (12%) | 25 (7%) | 12 (5%) |
| No preference | 481 (37%) | 82 (37%) | 147 (40%) | 49 (39%) | 123 (36%) | 80 (32%) |
| Venous | 285 (22%) | 34 (15%) | 75 (20%) | 30 (24%) | 91 (26%) | 55 (22%) |
| *Reasons for preferring a finger prick* | | | | | | |
| Quicker [n(%)] | 316 (69%) | 77 (77%) | 66 (55%) | 23 (70%) | 78 (74%) | 72 (71%) |
| More comfortable [n(%)] | 301 (65%) | 72 (72%) | 67 (56%) | 24 (73%) | 78 (74%) | 60 (59%) |
| Less painful [n(%)] | 194 (42%) | 54 (54%) | 33 (28%) | 15 (45%) | 46 (43%) | 46 (46%) |
| No bruises [n(%)] | 172 (37%) | 35 (35%) | 48 (40%) | 14 (42%) | 39 (37%) | 36 (36%) |
| I am used to it [n(%)] | 154 (33%) | 68 (68%) | 35 (29%) | 5 (15%) | 4 (4%) | 42 (42%) |
| Hard to find my vein [n(%)] | 133 (29%) | 23 (23%) | 30 (25%) | 16 (48%) | 34 (32%) | 30 (30%) |
| *No bleeding afterwards [n(%)]* | 83 (18%) | 11 (11%) | 26 (22%) | 6 (18%) | 17 (16%) | 23 (23%) |
| No muscle pain [n(%)] | 55 (12%) | 13 (13%) | 15 (13%) | 5 (15%) | 15 (14%) | 7 (7%) |
| Afraid of needles [n(%)] | 44 (10%) | 13 (13%) | 6 (5%) | 5 (15%) | 10 (9%) | 10 (10%) |
| Different reason [n(%)] | 12 (3%) | 2 (2%) | 5 (4%) | 1 (3%) | 1 (1%) | 3 (3%) |
| *Reasons for preferring venous sampling* | | | | | | |
| I am used to it [n(%)] | 163 (57%) | 18 (53%) | 41 (55%) | 14 (47%) | 53 (58%) | 37 (67%) |
| Less painful [n(%)] | 114 (40%) | 13 (38%) | 24 (32%) | 12 (40%) | 42 (46%) | 23 (42%) |
| Uncomfortable to use a finger prick [n(%)] | 107 (38%) | 18 (53%) | 30 (40%) | 11 (37%) | 27 (30%) | 21 (38%) |
| I don't want to do it myself [n(%)] | 35 (12%) | 8 (24%) | 10 (13%) | 1 (3%) | 10 (11%) | 6 (11%) |
| Different reason [n(%)] | 7 (2%) | 0 (0%) | 2 (3%) | 3 (10%) | 2 (2%) | 0 (0%) |

Additional Table 2. Reasons for being interested in Hem-Col versus not being interested in Hem-Col.

|  | Total | DM | CVD | CKD | TD | Mult |
| --- | --- | --- | --- | --- | --- | --- |
| Interested [n(%)] | 859 (66%) | 172 (77%) | 212 (57%) | 84 (66%) | 228 (66%) | 163 (66%) |
| Reasons |  |  |  |  |  |  |
| I can do it myself [n(%)] | 643 (75%) | 128 (74%) | 152 (72%) | 57 (68%) | 176 (77%) | 130 (80%) |
| Easier to plan into my schedule [n(%)] | 463 (54%) | 117 (68%) | 82 (39%) | 46 (55%) | 129 (57%) | 90 (55%) |
| Takes less time [n(%)] | 510 (60%) | 120 (70%) | 85 (40%) | 63 (75%) | 139 (61%) | 104 (64%) |
| No travelling needed [n(%)] | 467 (54%) | 102 (59%) | 90 (42%) | 56 (67%) | 121 (53%) | 98 (60%) |
| Different reason [n(%)] | 55 (6%) | 9 (5%) | 16 (8%) | 3 (4%) | 14 (6%) | 13 (8%) |
| Not interested [n(%)] | 196 (15%) | 19 (9%) | 64 (17%) | 17 (13%) | 58 (17%) | 38 (15%) |
| Reasons |  |  |  |  |  |  |
| Uncomfortable to do it myself [n(%)] | 103 (52%) | 10 (53%) | 37 (58%) | 7 (41%) | 33 (57%) | 16 (42%) |
| Fear to do it myself [n(%)] | 59 (30%) | 6 (32%) | 17 (27%) | 4 (24%) | 25 (43%) | 7 (18%) |
| I see it as a trip [n(%)] | 15 (8%) | 3 (16%) | 7 (11%) | 0 (0%) | 0 (0%) | 6 (16%) |
| Different reason [n(%)] | 67 (34%) | 6 (32%) | 24 (38%) | 7 (41%) | 12 (21%) | 18 (47%) |
| Indifferent [n(%)] | 256 (20%) | 31 (14%) | 93 (25%) | 26 (20%) | 59 (17%) | 47 (19%) |

Additional Table 3. Willingness to use Hem-Col among participants.

|  | Total | DM | CVD | CKD | TD | Mult |
| --- | --- | --- | --- | --- | --- | --- |
| Willingness to use hem-col [n(%)] | 933 (71%) | 181 (82%) | 230 (62%) | 96 (76%) | 257 (74%) | 169 (68%) |
| For all blood tests [n(%)] | 751 (81%) | 151 (68%) | 196 (53%) | 65 (51%) | 209 (61%) | 130 (52%) |
| Contribution willing to pay [mean(sd)] | €2.12 (4.44) | €1.91 (3.72) | €2.18 (4.21) | €2.32(5.90) | €2.75 (5.31) | €1.41 (2.78) |

## Representativeness of Survey respondents to the Dutch population.

A significant difference was found in the mean age between the responding patients and literature for the groups DM (51.8 versus 66.3 years; p<0.001), CVD (64.5 versus 76.5 years; p<0.001), CKD (51.0 versus 66.7 years; p<0.001) and TD (49.2 versus 58.9 years; p<0.001) [1-4]. A significant difference was found in gender between the responding patients and literature for the groups DM (male 38% versus 53%; p<0.001), CVD (male 57% versus 52%; p=0.02) and TD (male 8% versus 16%; p<0.001) [1-4]. Finally, a significant difference was found in distribution among Dutch provinces between the respondents and literature for the DM- (p=0.04), CVD- (p<0.001) and TD-patients (p<0.001) [5]. However, for CKD-patients no significant difference was found in gender (p=0.89) and distribution among Dutch provinces (p=0.43) [1-5]. Therefore, only the responding CKD-patients are representative of the Dutch population in terms of gender and province.

## References

1. Volksgezondheidenzorg.info. *Prevalentie diabetes in huisartsenpraktijk naar leeftijd en geslacht*. 2020 [cited 2020, 22 April]; Available from: <https://www.volksgezondheidenzorg.info/onderwerp/diabetes-mellitus/cijfers-context/huidige-situatie#node-prevalentie-diabetes-huisartsenpraktijk-naar-leeftijd-en-geslacht>.

2. de Boer, A., et al. *Hart- en vaatziekten in Nederland 2019*. 2019; Available from: <https://www.hartstichting.nl/getmedia/41cf66bf-2107-44d6-b2c3-739fc465ec73/cijferboek-hartstichting-hart-vaatziekten-nederland-2019-rp92.pdf>.

3. Flinterman, L., et al. *Vroege opsporing chronische nierschade*. 2019 [cited 2020, 4 April]; Available from: <https://nivel.nl/sites/default/files/bestanden/Vroege_opsporing_chronische_nierschade.pdf>.

4. van der Linden, M., et al. *Tweede nationale studie naar ziekten en verrichtingen in de huisartsenpraktijk.* . 2004 [cited 2020, 4 May]; Available from: <https://www.nivel.nl/sites/default/files/bestanden/ns2_rapport1.pdf>.

5. Metatopos. *Het koninkrijk der Nederlanden*. 2020 [cited 2020, 20 May]; Available from: <https://www.metatopos.eu/provincies_eu.php>.
