# Supplementary material for: The societal impact of implementing an at-home blood sampling device for chronic care patients: patient preferences and cost impact
Source: BMC Health Serv Res. 2022 Dec 15;22:1529. doi: 10.1186/s12913-022-08782-w (PMC9753888; doi:10.1186/s12913-022-08782-w)
Supplement: Supplementary file 3 — Additional file 3. Details of the cost-minimisation analysis. [file 12913_2022_8782_MOESM3_ESM.docx]

# Additional File 3: Details of the cost-minimisation analysis

1. Overview of model structure

An overview of the model structure is illustrated in Figure 1. The transition probabilities, found in Section 3.2, are unique for each disease, but the structure is identical for all diseases.

Additional Figure 1. Overview of model structure.

1. Summary of input parameters

Additional Table 1. Input probability parameters used in the patient-level monte carlo simulation.

| Parameters | Category | Probability | 95% CI | Distribution | Source |
| --- | --- | --- | --- | --- | --- |
| Probability of chronic disease | DM | 20.12% | 20.09% to 20.15% | Dirichlet | [1, 2] |
|  | CVD | 26.65% | 26.62% to 26.69% | Dirichlet | [3, 4] |
|  | CKD | 30.46% | 30.42% to 30.49% | Dirichlet | [5-7] |
|  | TD | 10.11% | 10.08% to 10.13% | Dirichlet | [8-11] |
|  | Mult | 12.66% | 12.64% to 12.69% | Dirichlet |  |
| Male gender | DM | 52.64% | 52.55% to 52.73% | Beta | [12] |
|  | CVD | 52.09% | 52.01% to 52.17% | Beta | [3] |
|  | CKD | 29.22% | 29.14% to 29.29% | Beta | [13] |
|  | TD | 15.50% | 15.33% to 15.67% | Beta | [14] |
|  | Mult | 42.95% | 42.86% to 43.04% | Beta |  |
| Population age distribution | 18-24 | 1.01% | 0.98% to 1.03% | Dirichlet | [12, 15] |
| (male with DM) | 25-34 | 1.62% | 1.58% to 1.65% | Dirichlet | [12, 15] |
|  | 35-44 | 3.91% | 3.87% to 3.96% | Dirichlet | [12, 15] |
|  | 45-54 | 12.81% | 12.73% to 12.90% | Dirichlet | [12, 15] |
|  | 55-64 | 23.62% | 23.52% to 23.73% | Dirichlet | [12, 15] |
|  | 65-74 | 31.92% | 31.81% to 32.04% | Dirichlet | [12, 15] |
|  | 75+ | 25.10% | 25.00% to 25.21% | Dirichlet | [12, 15] |
| Population age distribution | 18-24 | 1.24% | 1.21% to 1.27% | Dirichlet | [12, 15] |
| (female with DM) | 25-34 | 1.74% | 1.71% to 1.78% | Dirichlet | [12, 15] |
|  | 35-44 | 3.48% | 3.43% to 3.53% | Dirichlet | [12, 15] |
|  | 45-54 | 10.83% | 10.75% to 10.91% | Dirichlet | [12, 15] |
|  | 55-64 | 19.95% | 19.85% to 20.06% | Dirichlet | [12, 15] |
|  | 65-74 | 27.70% | 27.58% to 27.82% | Dirichlet | [12, 15] |
|  | 75+ | 35.06% | 34.94% to 35.19% | Dirichlet | [12, 15] |
| Population age distribution | 18-24 | 1.28% | 1.26% to 1.31% | Dirichlet | [3] |
| (male with CVD) | 25-34 | 2.14% | 2.11% to 2.17% | Dirichlet | [3] |
|  | 35-44 | 2.14% | 2.11% to 2.17% | Dirichlet | [3] |
|  | 45-54 | 2.47% | 2.44% to 2.50% | Dirichlet | [3] |
|  | 55-64 | 8.64% | 8.58% to 8.70% | Dirichlet | [3] |
|  | 65-74 | 19.14% | 19.05% to 19.22% | Dirichlet | [3] |
|  | 75+ | 64.20% | 64.09% to 64.30% | Dirichlet | [3] |
| Population age distribution | 18-24 | 1.70% | 1.67% to 1.73% | Dirichlet | [3] |
| (female with CVD) | 25-34 | 2.84% | 2.80% to 2.88% | Dirichlet | [3] |
|  | 35-44 | 2.84% | 2.80% to 2.88% | Dirichlet | [3] |
|  | 45-54 | 3.36% | 3.31% to 3.40% | Dirichlet | [3] |
|  | 55-64 | 8.72% | 8.66% to 8.79% | Dirichlet | [3] |
|  | 65-74 | 15.44% | 15.35% to 15.52% | Dirichlet | [3] |
|  | 75+ | 65.10% | 64.99% to 65.21% | Dirichlet | [3] |
| Population age distribution | 18-24 | 0.00% | 0.00% to 0.00% | Dirichlet | [13, 15] |
| (male with CKD) | 25-34 | 0.00% | 0.00% to 0.00% | Dirichlet | [13, 15] |
|  | 35-44 | 3.52% | 3.47% to 3.57% | Dirichlet | [13, 15] |
|  | 45-54 | 13.06% | 12.96% to 13.16% | Dirichlet | [13, 15] |
|  | 55-64 | 19.86% | 19.74% to 19.98% | Dirichlet | [13, 15] |
|  | 65-74 | 25.08% | 24.96% to 25.21% | Dirichlet | [13, 15] |
|  | 75+ | 38.47% | 38.33% to 38.62% | Dirichlet | [13, 15] |
| Population age distribution | 18-24 | 0.97% | 0.95% to 0.99% | Dirichlet | [13, 15] |
| (female with CKD) | 25-34 | 3.52% | 3.48% to 3.55% | Dirichlet | [13, 15] |
|  | 35-44 | 3.89% | 3.85% to 3.93% | Dirichlet | [13, 15] |
|  | 45-54 | 12.49% | 12.43% to 12.55% | Dirichlet | [13, 15] |
|  | 55-64 | 19.67% | 19.60% to 19.75% | Dirichlet | [13, 15] |
|  | 65-74 | 26.54% | 26.46% to 26.63% | Dirichlet | [13, 15] |
|  | 75+ | 32.91% | 32.83% to 33.00% | Dirichlet | [13, 15] |
| Population age distribution | 18-24 | 2.37% | 2.19% to 2.55% | Dirichlet | [14, 15] |
| (male with TD) | 25-34 | 5.44% | 5.17% to 5.71% | Dirichlet | [14, 15] |
|  | 35-44 | 5.44% | 5.17% to 5.71% | Dirichlet | [14, 15] |
|  | 45-54 | 18.64% | 18.18% to 19.11% | Dirichlet | [14, 15] |
|  | 55-64 | 18.64% | 18.18% to 19.11% | Dirichlet | [14, 15] |
|  | 65-74 | 27.35% | 26.82% to 27.88% | Dirichlet | [14, 15] |
|  | 75+ | 22.11% | 21.62% to 22.60% | Dirichlet | [14, 15] |
| Population age distribution | 18-24 | 2.23% | 2.16% to 2.31% | Dirichlet | [14, 15] |
| (female with TD) | 25-34 | 8.20% | 8.06% to 8.34% | Dirichlet | [14, 15] |
|  | 35-44 | 8.20% | 8.06% to 8.34% | Dirichlet | [14, 15] |
|  | 45-54 | 20.07% | 19.86% to 20.27% | Dirichlet | [14, 15] |
|  | 55-64 | 20.07% | 19.86% to 20.27% | Dirichlet | [14, 15] |
|  | 65-74 | 22.59% | 22.38% to 22.80% | Dirichlet | [14, 15] |
|  | 75+ | 18.64% | 18.45% to 18.84% | Dirichlet | [14, 15] |
| Population age distribution | 18-24 | 0.91% | 0.88% to 0.94% | Dirichlet |  |
| (male with mult) | 25-34 | 1.52% | 1.48% to 1.55% | Dirichlet |  |
|  | 35-44 | 3.08% | 3.04% to 3.13% | Dirichlet |  |
|  | 45-54 | 8.53% | 8.45% to 8.61% | Dirichlet |  |
|  | 55-64 | 16.27% | 16.16% to 16.37% | Dirichlet |  |
|  | 65-74 | 24.80% | 24.67% to 24.92% | Dirichlet |  |
|  | 75+ | 44.89% | 44.75% to 45.03% | Dirichlet |  |
| Population age distribution | 18-24 | 1.32% | 1.29% to 1.35% | Dirichlet |  |
| (female with Mult) | 25-34 | 3.20% | 3.16% to 3.25% | Dirichlet |  |
|  | 35-44 | 3.74% | 3.70% to 3.79% | Dirichlet |  |
|  | 45-54 | 9.87% | 9.79% to 9.94% | Dirichlet |  |
|  | 55-64 | 16.51% | 16.42% to 16.61% | Dirichlet |  |
|  | 65-74 | 23.28% | 23.17% to 23.38% | Dirichlet |  |
|  | 75+ | 42.08% | 41.95% to 42.20% | Dirichlet |  |
| Location for DM 18-24 | Hospital | 70.73% | 56.80% to 84.66% | Dirichlet | Survey |
|  | Service phlebotomy center | 29.27% | 15.34% to 43.20% | Dirichlet | Survey |
|  | GP's office | 0.00% | 0.00% to 0.00% | Dirichlet | Survey |
|  | At home | 0.00% | 0.00% to 0.00% | Dirichlet | Survey |
| Location for DM 25-34 | Hospital | 72.97% | 58.66% to 87.28% | Dirichlet | Survey |
|  | Service phlebotomy center | 24.32% | 10.50% to 38.15% | Dirichlet | Survey |
|  | GP's office | 2.70% | 0.00% to 7.93% | Dirichlet | Survey |
|  | At home | 0.00% | 0.00% to 0.00% | Dirichlet | Survey |
| Location for DM 35-44 | Hospital | 64.00% | 45.18% to 82.82% | Dirichlet | Survey |
|  | Service phlebotomy center | 36.00% | 17.18% to 54.82% | Dirichlet | Survey |
|  | GP's office | 0.00% | 0.00% to 0.00% | Dirichlet | Survey |
|  | At home | 0.00% | 0.00% to 0.00% | Dirichlet | Survey |
| Location for DM 45-54 | Hospital | 67.50% | 52.98% to 82.02% | Dirichlet | Survey |
|  | Service phlebotomy center | 25.00% | 11.58% to 38.42% | Dirichlet | Survey |
|  | GP's office | 2.50% | 0.00% to 7.34% | Dirichlet | Survey |
|  | At home | 5.00% | 0.00% to 11.75% | Dirichlet | Survey |
| Location for DM 55-64 | Hospital | 43.75% | 26.56% to 60.94% | Dirichlet | Survey |
|  | Service phlebotomy center | 40.63% | 23.61% to 57.64% | Dirichlet | Survey |
|  | GP's office | 12.50% | 1.04% to 23.96% | Dirichlet | Survey |
|  | At home | 3.13% | 0.00% to 9.15% | Dirichlet | Survey |
| Location for DM 65-75+ | Hospital | 29.79% | 16.71% to 42.86% | Dirichlet | Survey |
|  | Service phlebotomy center | 59.57% | 45.54% to 73.60% | Dirichlet | Survey |
|  | GP's office | 6.38% | 0.00% to 13.37% | Dirichlet | Survey |
|  | At home | 4.26% | 0.00% to 10.03% | Dirichlet | Survey |
| Location for CVD 18-54 | Hospital | 36.76% | 25.30% to 48.23% | Dirichlet | Survey |
|  | Service phlebotomy center | 51.47% | 39.59% to 63.35% | Dirichlet | Survey |
|  | GP's office | 8.82% | 2.08% to 15.57% | Dirichlet | Survey |
|  | At home | 2.94% | 0.00% to 6.96% | Dirichlet | Survey |
| Location for CVD 55-64 | Hospital | 39.58% | 29.80% to 49.37% | Dirichlet | Survey |
|  | Service phlebotomy center | 42.71% | 32.81% to 52.60% | Dirichlet | Survey |
|  | GP's office | 11.46% | 5.09% to 17.83% | Dirichlet | Survey |
|  | At home | 6.25% | 1.41% to 11.09% | Dirichlet | Survey |
| Location for CVD 65-74 | Hospital | 38.36% | 30.47% to 46.24% | Dirichlet | Survey |
|  | Service phlebotomy center | 50.00% | 41.89% to 58.11% | Dirichlet | Survey |
|  | GP's office | 8.22% | 3.76% to 12.67% | Dirichlet | Survey |
|  | At home | 3.42% | 0.47% to 6.37% | Dirichlet | Survey |
| Location for CVD 75+ | Hospital | 30.51% | 18.76% to 42.26% | Dirichlet | Survey |
|  | Service phlebotomy center | 42.37% | 29.76% to 54.98% | Dirichlet | Survey |
|  | GP's office | 8.47% | 1.37% to 15.58% | Dirichlet | Survey |
|  | At home | 18.64% | 8.71% to 28.58% | Dirichlet | Survey |
| Location for CKD 18-34 | Hospital | 94.59% | 87.31% to 88.88% | Dirichlet | Survey |
|  | Service phlebotomy center | 2.70% | 0.00% to 7.93% | Dirichlet | Survey |
|  | GP's office | 2.70% | 0.00% to 7.93% | Dirichlet | Survey |
|  | At home | 0.00% | 0.00% to 0.00% | Dirichlet | Survey |
| Location for CKD 35-44 | Hospital | 83.33% | 68.42% to 98.24% | Dirichlet | Survey |
|  | Service phlebotomy center | 16.67% | 1.76% to 31.58% | Dirichlet | Survey |
|  | GP's office | 0.00% | 0.00% to 0.00% | Dirichlet | Survey |
|  | At home | 0.00% | 0.00% to 0.00% | Dirichlet | Survey |
| Location for CKD 45-54 | Hospital | 77.78% | 62.10% to 93.46% | Dirichlet | Survey |
|  | Service phlebotomy center | 18.52% | 3.87% to 33.17% | Dirichlet | Survey |
|  | GP's office | 3.70% | 0.00% to 10.83% | Dirichlet | Survey |
|  | At home | 0.00% | 0.00% to 0.00% | Dirichlet | Survey |
| Location for CKD 55-75+ | Hospital | 74.36% | 60.65% to 88.06% | Dirichlet | Survey |
|  | Service phlebotomy center | 20.51% | 7.84% to 33.19% | Dirichlet | Survey |
|  | GP's office | 2.56% | 0.00% to 7.52% | Dirichlet | Survey |
|  | At home | 2.56% | 0.00% to 7.52% | Dirichlet | Survey |
| Location for TD 18-34 | Hospital | 57.63% | 45.02% to 70.24% | Dirichlet | Survey |
|  | Service phlebotomy center | 33.90% | 21.82% to 45.98% | Dirichlet | Survey |
|  | GP's office | 8.47% | 1.37% to 15.58% | Dirichlet | Survey |
|  | At home | 0.00% | 0.00% to 0.00% | Dirichlet | Survey |
| Location for TD 35-44 | Hospital | 43.55% | 31.21% to 55.89% | Dirichlet | Survey |
|  | Service phlebotomy center | 48.39% | 35.95% to 60.83% | Dirichlet | Survey |
|  | GP's office | 8.06% | 1.29% to 14.84% | Dirichlet | Survey |
|  | At home | 0.00% | 0.00% to 0.00% | Dirichlet | Survey |
| Location for TD 45-54 | Hospital | 46.22% | 37.26% to 55.18% | Dirichlet | Survey |
|  | Service phlebotomy center | 47.06% | 38.09% to 56.03% | Dirichlet | Survey |
|  | GP's office | 6.72% | 2.22% to 11.22% | Dirichlet | Survey |
|  | At home | 0.00% | 0.00% to 0.00% | Dirichlet | Survey |
| Location for TD 55-64 | Hospital | 42.50% | 31.67% to 53.33% | Dirichlet | Survey |
|  | Service phlebotomy center | 41.25% | 30.46% to 52.04% | Dirichlet | Survey |
|  | GP's office | 16.25% | 8.17% to 24.33% | Dirichlet | Survey |
|  | At home | 0.00% | 0.00% to 0.00% | Dirichlet | Survey |
| Location for TD 65-75+ | Hospital | 36.00% | 17.18% to 54.82% | Dirichlet | Survey |
|  | Service phlebotomy center | 48.00% | 28.42% to 67.58% | Dirichlet | Survey |
|  | GP's office | 16.00% | 1.63% to 30.37% | Dirichlet | Survey |
|  | At home | 0.00% | 0.00% to 0.00% | Dirichlet | Survey |
| Location for Mult 18-44 | Hospital | 68.57% | 53.19% to 83.95% | Dirichlet | Survey |
|  | Service phlebotomy center | 28.57% | 13.60% to 43.54% | Dirichlet | Survey |
|  | GP's office | 2.86% | 0.00% to 8.38% | Dirichlet | Survey |
|  | At home | 0.00% | 0.00% to 0.00% | Dirichlet | Survey |
| Location for Mult 45-54 | Hospital | 56.82% | 42.18% to 71.45% | Dirichlet | Survey |
|  | Service phlebotomy center | 38.64% | 24.25% to 53.02% | Dirichlet | Survey |
|  | GP's office | 4.55% | 0.00% to 10.70% | Dirichlet | Survey |
|  | At home | 0.00% | 0.00% to 0.00% | Dirichlet | Survey |
| Location for Mult 55-64 | Hospital | 66.67% | 54.74% to 78.59% | Dirichlet | Survey |
|  | Service phlebotomy center | 26.67% | 15.48% to 37.86% | Dirichlet | Survey |
|  | GP's office | 6.67% | 0.35% to 12.98% | Dirichlet | Survey |
|  | At home | 0.00% | 0.00% to 0.00% | Dirichlet | Survey |
| Location for Mult 65-74 | Hospital | 37.33% | 26.39% to 48.28% | Dirichlet | Survey |
|  | Service phlebotomy center | 53.33% | 42.04% to 64.62% | Dirichlet | Survey |
|  | GP's office | 6.67% | 1.02% to 12.31% | Dirichlet | Survey |
|  | At home | 2.67% | 0.00% to 6.31% | Dirichlet | Survey |
| Location for Mult 75+ | Hospital | 32.35% | 16.63% to 48.08% | Dirichlet | Survey |
|  | Service phlebotomy center | 47.06% | 30.28% to 63.84% | Dirichlet | Survey |
|  | GP's office | 8.82% | 0.00% to 18.36% | Dirichlet | Survey |
|  | At home | 11.76% | 0.93% to 22.59% | Dirichlet | Survey |
| Dependency on others for DM | 18-24 | 17.07% | 5.56% to 28.59% | Beta | Survey |
|  | 25-34 | 13.51% | 2.50% to 24.53% | Beta | Survey |
|  | 35-44 | 28.00% | 10.40% to 45.60% | Beta | Survey |
|  | 45-54 | 10.00% | 0.70% to 19.30% | Beta | Survey |
|  | 55-64 | 18.75% | 5.23% to 32.27% | Beta | Survey |
|  | 65-75+ | 17.50% | 6.64% to 28.36% | Beta | Survey |
| Dependency on others for CVD | 18-54 | 22.06% | 12.20% to 31.91% | Beta | Survey |
|  | 55-64 | 14.58% | 7.52% to 21.64% | Beta | Survey |
|  | 65-74 | 7.53% | 3.25% to 11.82% | Beta | Survey |
|  | 75+ | 13.56% | 4.82% to 22.30% | Beta | Survey |
| Dependency on others for CKD | 18-34 | 15.63% | 3.93% to 27.32% | Beta | Survey |
|  | 35-44 | 20.83% | 4.59% to 37.08% | Beta | Survey |
|  | 45-54 | 14.81% | 1.41% to 28.21% | Beta | Survey |
|  | 55-75+ | 21.88% | 8.90% to 34.85% | Beta | Survey |
| Dependency on others for TD | 18-34 | 18.00% | 8.20% to 27.80% | Beta | Survey |
|  | 35-44 | 12.90% | 4.56% to 21.25% | Beta | Survey |
|  | 45-54 | 12.61% | 6.64% to 18.57% | Beta | Survey |
|  | 55-64 | 21.25% | 12.29% to 30.21% | Beta | Survey |
|  | 64-75+ | 19.05% | 3.65% to 34.44% | Beta | Survey |
| Dependency on others for Mult | 18-44 | 40.00% | 23.77% to 56.23% | Beta | Survey |
|  | 45-54 | 20.45% | 8.54% to 32.37% | Beta | Survey |
|  | 55-64 | 20.00% | 9.88% to 30.12% | Beta | Survey |
|  | 65-74 | 18.67% | 9.85% to 27.49% | Beta | Survey |
|  | 75+ | 20.59% | 7.00% to 34.18% | Beta | Survey |
| Willing to use hem-col DM | 18-24 | 89.02% | 79.46% to 98.59% | Beta | Survey |
|  | 25-34 | 81.08% | 68.46% to 93.70% | Beta | Survey |
|  | 35-44 | 88.00% | 75.26% to 99.74% | Beta | Survey |
|  | 45-54 | 87.50% | 77.25% to 97.75% | Beta | Survey |
|  | 55-64 | 90.63% | 80.53% to 99.72% | Beta | Survey |
|  | 65-75+ | 74.47% | 62.00% to 86.93% | Beta | Survey |
| Willing to use hem-col CVD | 18-54 | 77.21% | 67.31% to 87.10% | Beta | Survey |
|  | 55-64 | 68.75% | 59.48% to 78.02% | Beta | Survey |
|  | 65-74 | 67.47% | 59.87% to 75.07% | Beta | Survey |
|  | 75+ | 70.34% | 58.68% to 81.99% | Beta | Survey |
| Willing to use hem-col CKD | 18-34 | 78.38% | 65.11% to 91.64% | Beta | Survey |
|  | 35-44 | 85.42% | 71.58% to 99.25% | Beta | Survey |
|  | 45-54 | 74.07% | 57.54% to 90.60% | Beta | Survey |
|  | 55-75+ | 70.51% | 56.38% to 84.64% | Beta | Survey |
| Willing to use hem-col TD | 18-34 | 72.03% | 60.68% to 83.39% | Beta | Survey |
|  | 35-44 | 79.84% | 69.85% to 89.83% | Beta | Survey |
|  | 45-54 | 73.11% | 65.14% to 81.08% | Beta | Survey |
|  | 55-64 | 76.25% | 66.92% to 85.58% | Beta | Survey |
|  | 64-75+ | 70.00% | 52.39% to 87.61% | Beta | Survey |
| Willing to use hem-col Mult | 18-44 | 85.71% | 74.12% to 97.31% | Beta | Survey |
|  | 45-54 | 78.41% | 66.25% to 90.57% | Beta | Survey |
|  | 55-64 | 81.67% | 71.88% to 91.46% | Beta | Survey |
|  | 65-74 | 70.00% | 59.63% to 80.37% | Beta | Survey |
|  | 75+ | 60.29% | 43.85% to 76.74% | Beta | Survey |
| CI = confidence interval, CKD = chronic kidney disease, CVD = cardiovascular diseases, DM = diabetes mellitus, GP = general practitioner, TD = thyroid diseases. | | | | | |

Additional Table 2. Input parameters used in the patient-level monte carlo simulation.

| Parameters | Category | Value | 95% CI | Distribution | Source |
| --- | --- | --- | --- | --- | --- |
| Amount of phlebotomy appoint- | 18-24 | 3.00 | 2.54 to 3.46 | Gamma | Survey |
| ments per year for DM | 25-34 | 3.59 | 3.05 to 4.14 | Gamma | Survey |
|  | 35-44 | 3.28 | 2.88 to 3.68 | Gamma | Survey |
|  | 45-54 | 3.83 | 3.37 to 4.28 | Gamma | Survey |
|  | 55-64 | 3.22 | 2.71 to 3.73 | Gamma | Survey |
|  | 65-75+ | 3.91 | 1.8 to 6.01 | Gamma | Survey |
| Amount of phlebotomy appoint- | 18-34 | 7.23 | 4.91 to 9.56 | Gamma | Survey |
| ments per year for CKD | 35-44 | 5.63 | 4.34 to 6.91 | Gamma | Survey |
|  | 45-54 | 4.41 | 3.7 to 5.12 | Gamma | Survey |
|  | 55-75+ | 5.78 | 4.45 to 7.12 | Gamma | Survey |
| Amount of phlebotomy appoint- | 18-34 | 5.05 | 3.67 to 6.43 | Gamma | Survey |
| ments per year for TD | 35-44 | 5.02 | 4.17 to 5.86 | Gamma | Survey |
|  | 45-54 | 4.43 | 3.87 to 4.99 | Gamma | Survey |
|  | 55-64 | 3.88 | 3.22 to 4.53 | Gamma | Survey |
|  | 64-75+ | 2.60 | 1.34 to 3.86 | Gamma | Survey |
| Amount of phlebotomy appoint- | 18-44 | 4.28 | 3.37 to 5.18 | Gamma | Survey |
| ments per year for Mult | 45-54 | 5.00 | 3.81 to 6.19 | Gamma | Survey |
|  | 55-64 | 4.98 | 3.88 to 6.08 | Gamma | Survey |
|  | 65-74 | 4.41 | 3.32 to 5.5 | Gamma | Survey |
|  | 75+ | 7.91 | 1.14 to 14.69 | Gamma | Survey |
| Amount of phlebotomy appoint- | 18-54 | 4.52 | 3.18 to 5.85 | Gamma | Survey |
| ments per year for CVD | 55-64 | 3.66 | 2.31 to 5.01 | Gamma | Survey |
|  | 65-74 | 3.51 | 2.54 to 4.48 | Gamma | Survey |
|  | 75+ | 6.24 | 3.89 to 8.59 | Gamma | Survey |
| Time spent at the hospital in hours | DM | 1.22 | 1.14 to 1.31 | Gamma | Survey |
|  | CVD | 1.15 | 1.06 to 1.23 | Gamma | Survey |
|  | CKD | 1.39 | 1.28 to 1.5 | Gamma | Survey |
|  | TD | 1.11 | 1.03 to 1.19 | Gamma | Survey |
|  | Mult | 1.34 | 1.22 to 1.45 | Gamma | Survey |
| Time spent at the service phleboto- | DM | 0.85 | 0.77 to 0.93 | Gamma | Survey |
| my center in hours | CVD | 0.82 | 0.77 to 0.87 | Gamma | Survey |
|  | CKD | 0.75 | 0.62 to 0.88 | Gamma | Survey |
|  | TD | 0.86 | 0.8 to 0.93 | Gamma | Survey |
|  | Mult | 0.89 | 0.81 to 0.97 | Gamma | Survey |
| Time spent at the GP's office in | DM | 0.83 | 0.67 to 1 | Gamma | Survey |
| hours | CVD | 0.65 | 0.57 to 0.72 | Gamma | Survey |
|  | CKD | 0.83 | 0.51 to 1.16 | Gamma | Survey |
|  | TD | 0.80 | 0.68 to 0.92 | Gamma | Survey |
|  | Mult | 0.83 | 0.65 to 1.02 | Gamma | Survey |
| Time spent at home in hours | DM | 0.80 | 0.21 to 1.39 | Gamma | Survey |
|  | CVD | 0.46 | 0.34 to 0.58 | Gamma | Survey |
|  | CKD | 1.00 | 0 | Gamma | Survey |
|  | TD | 1.00 | 0 | Gamma | Survey |
|  | Mult | 0.42 | 0.25 to 0.58 | Gamma | Survey |
| Time spent with hem-col in hours | | 0.54 | 0.28 to 0.81 | Gamma | [16] |
| CI = confidence interval, CKD = chronic kidney disease, CVD = cardiovascular diseases, DM = diabetes mellitus, GP = general practitioner, TD = thyroid diseases. | | | | | |

Additional Table 3. Input cost parameters used in the patient-level monte carlo simulation.

| Parameters | Category | Cost | 95% CI* | Distribution | Source |
| --- | --- | --- | --- | --- | --- |
| Costs venous | Hospital | € 9.04 | €8.08 to €9.99 | Gamma | [17-21] |
|  | Service phlebotomy center | € 15.34 | €14.09 to €16.60 | Gamma | [22-31] |
|  | GP's office | € 18.13 | €17.92 to €18.34 | Gamma | [22-31] |
|  | At home | € 25.16 | €19.36 to €30.96 | Gamma | [22-31] |
| Costs Hem-col | Hem-col | € 20.42 | €10.42 to €30.43 | Gamma | [16, 32, 33] |
|  | Extra tube | € 1.95 | €0.99 to €2.91 | Gamma | [16] |
| Waste processing per tube | Venous | € 0.01223 | €0.0062 to €0.0182 | Gamma | [34-38] |
|  | Hem-col | € 0.00679 | €0.0035 to €0.0101 | Gamma | [34-39] |
| Travel costs^1^ | Hospital | € 6.08 | €3.10 to €9.07 | Gamma | [40] |
|  | Service phlebotomy center | € 1.02 | €0.52 to €1.52 | Gamma | [40] |
|  | GP's office | € 0.45 | €0.23 to €0.67 | Gamma | [40] |
|  | Hem-col | € 1.02 | €0.52 to €1.52 | Gamma | [40-42] |
| Productivity loss costs per hour per male patient | 18-24 | € 40.74 | €20.78 to €60.70 | Gamma | [40] |
|  | 25-34 | € 40.74 | €20.78 to €60.70 | Gamma | [40] |
|  | 35-44 | € 40.74 | €20.78 to €60.70 | Gamma | [40] |
|  | 45-54 | € 40.74 | €20.78 to €60.70 | Gamma | [40] |
|  | 55-64 | € 40.74 | €20.78 to €60.70 | Gamma | [40] |
|  | 65-74 | € 4.91 | €2.51 to €7.32 | Gamma | [40, 43] |
|  | 75+ | € 0.73 | €0.37 to €1.09 | Gamma | [40, 43] |
| Productivity loss costs per hour per female patient | 18-24 | € 33.97 | €17.32 to €50.61 | Gamma | [40] |
|  | 25-34 | € 33.97 | €17.32 to €50.61 | Gamma | [40] |
|  | 35-44 | € 33.97 | €17.32 to €50.61 | Gamma | [40] |
|  | 45-54 | € 33.97 | €17.32 to €50.61 | Gamma | [40] |
|  | 55-64 | € 33.97 | €17.32 to €50.61 | Gamma | [40] |
|  | 65-74 | € 4.10 | €2.09 to €6.10 | Gamma | [40, 43] |
|  | 75+ | € 0.61 | €0.31 to €0.91 | Gamma | [40, 43] |
| Costs informal care giver per hour | | € 15.05 | €7.68 to €22.42 | Gamma | [40] |
| GP = general practitioner.  * 95% CI is based on an assumed standard error of 25%, except for the costs of venous sampling.  ^1^ Parking costs were added to the traveling costs when traveling to the hospital since almost all hospitals in the Netherlands have a paid parking lot . With Hem-Col, traveling costs were seen as the costs associated with mailing the sample to the laboratory. This was calculated by looking at the maximum distance to a mailbox (derived from PostNL, the Dutch postal network [29]) and the average cost per kilometer when traveling by car or public transport. | | | | | |

1. Scenario analysis

A scenario analysis was performed to investigate what the cost of the Hem-Col device should be to ensure that the overall phlebotomy cost when implementing Hem-col, remains comparable to current practice (venipuncture on location). It was found that when the price of Hem-col is reduced to €12.81 (opposed to the current cost of €22.42), the phlebotomy costs will remain similar to that of venipuncture on location. Monte Carlo simulations were performed for a probabilistic analysis (PA), using 10,000 iterations of 100,000 hypothetical chronic care patients with one of the four chronic diseases or multiple diseases. The average outcomes of the PA samples are presented in Figure 2.

Additional Figure 2. Analysis results of 100,000 hypothetical patients per year.

1. References

1. Diabetes Fonds. *Diabetes in cijfers*. [cited 2020, 18 February]; Available from: <https://www.diabetesfonds.nl/over-diabetes/diabetes-in-het-algemeen/diabetes-in-cijfers>.

2. Rijksinstituut voor Volksgezondheid en Milieu RIVM. *Aandoeningen: Welke aandoeningen hebben we in de toekomst?* 2018 [cited 2020, 12 March]; Available from: <https://www.vtv2018.nl/aandoeningen>.

3. de Boer, A., et al. *Hart- en vaatziekten in Nederland 2019*. 2019; Available from: <https://www.hartstichting.nl/getmedia/41cf66bf-2107-44d6-b2c3-739fc465ec73/cijferboek-hartstichting-hart-vaatziekten-nederland-2019-rp92.pdf>.

4. Hartstichting. *Cijfers hart- en vaatziekten in Nederland*. 2019; Available from: <https://nederlandsehartregistratie.nl/hartenvaatcijfers/wp-content/uploads/2019/12/Infografic-Cijfers-HVZ-2019.pdf>.

5. Nieren.nl. *Hoe vaak komt chronische nierschade voor?* 2018 [cited 2020, 31 March]; Available from: <https://www.nieren.nl/bibliotheek/28-feiten-en-cijfers/37-hoe-vaak-komt-chronische-nierschade-voor>.

6. Nieratlas. *Prevalentie*. [cited 2020, 31 March]; Available from: <https://nieratlas.nl/thema/aantalpatienten/prevalentie>.

7. Gansevoort, R. and B. van der Poel. *Chronische nierschade: hoe vaak, stadia en risico's* 2018 [cited 2020, 6 April]; Available from: <https://www.nierstichting.nl/media/filer_public/e4/07/e40798b6-40e1-4d54-b9c0-9f0367e78cf7/factsheet_3_chronische_nierschade.pdf>.

8. Schildklier Organisatie Nederland SON. *Te trage schildklier*. [cited 2020, 20 April]; Available from: <https://schildklier.nl/schildklieraandoeningen/te-trage-schildklier/>.

9. Thuisarts.nl. *Mijn schildklier werkt te snel*. [cited 2020, 20 April]; Available from: <https://www.thuisarts.nl/schildklierafwijking/mijn-schildklier-werkt-te-snel>.

10. Integraal Kankercentrum Nederland (IKNL). *Prevalentie schildklierkanker*. [cited 2020, 20 April]; Available from: <https://www.iknl.nl/kankersoorten/schildklierkanker/registratie/prevalentie>.

11. Gezondheidsplein. *Ziekte van Graves*. [cited 2020, 20 April]; Available from: <https://www.gezondheidsplein.nl/aandoeningen/ziekte-van-graves/item39561>.

12. Volksgezondheidenzorg.info. *Prevalentie diabetes in huisartsenpraktijk naar leeftijd en geslacht*. 2020 [cited 2020, 22 April]; Available from: <https://www.volksgezondheidenzorg.info/onderwerp/diabetes-mellitus/cijfers-context/huidige-situatie#node-prevalentie-diabetes-huisartsenpraktijk-naar-leeftijd-en-geslacht>.

13. Flinterman, L., et al. *Vroege opsporing chronische nierschade*. 2019 [cited 2020, 4 April]; Available from: <https://nivel.nl/sites/default/files/bestanden/Vroege_opsporing_chronische_nierschade.pdf>.

14. van der Linden, M., et al. *Tweede nationale studie naar ziekten en verrichtingen in de huisartsenpraktijk.* . 2004 [cited 2020, 4 May]; Available from: <https://www.nivel.nl/sites/default/files/bestanden/ns2_rapport1.pdf>.

15. Centraal Bureau voor de Statistiek. *Bevolingspyramide*. 2019 [cited 2020, 4 May]; Available from: <https://www.cbs.nl/nl-nl/visualisaties/bevolkingspiramide>.

16. Personal communication with D. Poland. *Labonovum, Limmen, The Netherlands*.

17. Sint Antonius ziekenhuis. *Passantenprijslijst 2019*. [cited 2020, 7 June]; Available from: <https://www.antoniusziekenhuis.nl/sites/default/files/offerte_passanten_20191001-20191231-update_dd_20191119.pdf>.

18. Franciscus Gasthuis & Vlietland. *Passantenprijslijst 2020*. [cited 2020, 7 June]; Available from: <https://www.franciscus.nl/uploads/tekstblok/publicatie_passantenprijslijst_2020_dotovp.pdf>.

19. Elisabeth Tweesteden Ziekenhuis (ETZ). *Standaard prijslijst DBC-zorgproducten 2020*. [cited 2020, 7 June]; Available from: <https://www.etz.nl/ETZWebsite/files/5c/5ca3cb35-694b-47c2-ae25-5be8567ddfb8.pdf>.

20. Catharina Ziekenhuis. *Prijslijst DBC zorgproducten 2018*. [cited 2020, 7 June]; Available from: <https://www.catharinaziekenhuis.nl/files/Patient/Praktische_info/Tarieven/Tarievenlijst_2018_met_openingsdatum_tussen_01-01-2018_tm_31-12-2018.pdf>.

21. Martini Ziekenhuis. *Tarieven DBC zorgproducten 2019*. [cited 2020, 7 June]; Available from: <https://www.martiniziekenhuis.nl/Documents/Kosten/Tarieven%20DBC%20zorgproducten%202019.pdf>.

22. Atalmedial. *Maximumtarieven laboratoriumonderzoek*. [cited 2020, 18 March]; Available from: <https://www.atalmedial.nl/wp-content/uploads/2020/01/Atalmedial-basistarieven-ELD-2020.-Blad1.pdf>.

23. Salt. *Tarieven*. [cited 2020, 18 March]; Available from: <https://www.salt.nl/tarieven/>.

24. Isala. *Diagnostiek tarieven 2018 aanvrager huisarts*. [cited 2020, 18 March]; Available from: <https://www.isala.nl/media/25869/tarieven-diagnostiek-aanvrager-huisarts-2018.pdf>.

25. Saltro. *Informatie over de tarieven*. [cited 2020, 18 March]; Available from: <https://saltro.nl/informatie-over-tarieven>.

26. Star-shl. *Tarieven*. [cited 2020, 18 March]; Available from: <https://www.star-shl.nl/patient/tarieven/>.

27. Result laboratorium. *DBC-zorgproduct medische omschrijving*. [cited 2020, 18 March]; Available from: <https://resultlaboratorium.nl/wp-content/uploads/2020/02/Tarieven-diagnostiek-2020.pdf>.

28. Sanquin. *Tarieven 2020*. [cited 2020, 18 March]; Available from: <https://www.sanquin.org/nl/producten-en-diensten/diagnostiek/tarieflijst/index>.

29. Reinier Haga. *Tarieven laboratoriumdiagnostiek 2020*. [cited 2020, 18 March]; Available from: <https://www.rhmdc.nl/patienten/prijzen-laboratoriumtesten/>.

30. Diagnostiek Voor U. *Tarieven*. [cited 2020, 12 March]; Available from: <https://www.diagnostiekvooru.nl/tarieven>.

31. Medlon. *Medlon tarieven 2020*. [cited 2020, 18 March]; Available from: <https://www.medlon.nl/patienten/medlon-tarieven-2020/>.

32. Post NL. *Tarieven*. [cited 2020, 8 April]; Available from: <https://www.postnl.nl/tarieven>.

33. Post NL. *BTW-tarieven*. [cited 2020, 8 April]; Available from: <https://www.postnl.nl/klantenservice/zakelijk/facturen/belasting/btw-tarief/>.

34. Engels logistiek. *Veilige afvalinzameling in de zorgsector*. [cited 2020, 19 March]; Available from: <https://www.engelslogistiek.nl/pub/media/wysiwyg/Brochures/1902-Zorgsector.pdf>.

35. Erasmus MC. *Afval informatiemap Erasmus MC*. 2014 [cited 2020, 23 April]; Available from: [www.tenderned.nl](https://d.docs.live.net/c6226edc62bc0d4f/PhD/Paper%205.%20HEA/DRAFTS/www.tenderned.nl)›map›akid›false›actie›cid.

36. Renewi. *Risicohoudend medisch afval*. 2017 [cited 2020, 29 April]; Available from: [www.renewi.com](https://d.docs.live.net/c6226edc62bc0d4f/PhD/Paper%205.%20HEA/DRAFTS/www.renewi.com)›gevaarlijk-afval.

37. Personal communication with Renewi. *Prijs per 1000 kg specifiek ziekenhuisafval*.

38. Personal communication with Suez. *Prijs per 1000 kg specifiek ziekenhuisafval*.

39. Renewi. *660 liter rolcontainer voor restafval*. [cited 2020, 29 April]; Available from: <https://www.renewi.com/nl-nl/container-huren/660-liter-rolcontainer-voor-restafval--Package-00556>.

40. Hakkaart-van Roijen, L., et al., *Handleiding voor kostenonderzoek, methoden en standaard kostprijzen voor economische evaluaties in de gezondheidszorg [Manual for Cost Research, Methods and Standard Cost Prices for Economic Evaluations in Health Care].* 2010.

41. Post NL. *Brievenbussen*. [cited 2020, 12 May]; Available from: <https://www.postnl.nl/versturen/brief-of-kaart-versturen/hoe-verstuur-ik-een-brief-of-kaart/brievenbussen/>.

42. Centraal Bureau voor de Statistiek. *Fietsend achterop*. [cited 2020, 18 May]; Available from: [www.cbs.nl](https://d.docs.live.net/c6226edc62bc0d4f/PhD/Paper%205.%20HEA/DRAFTS/www.cbs.nl)›documents›index1294.

43. Centraal Bureau voor de Statistiek. *Ruim een kwart miljoen werkende 65-plussers*. 2019 [cited 2020, 1 June]; Available from: <https://www.cbs.nl/nl-nl/nieuws/2019/38/ruim-een-kwart-miljoen-werkende-65-plussers>.
